# Appearance-based computer vision pipeline for multi-animal monitoring of canine activity, behavior and clinical observations

**DOI:** 10.3389/ftox.2026.1758963

**Published:** 2026-02-18

**Authors:** Eline Eberhardt, Jef Plochaet, Tanguy Ophoff, Floris De Feyter, Sarah De Landtsheer, Greet Teuns, Maarten Vergauwen, Bianca Feyen, Toon Goedemé, Ivan Kopljar

**Affiliations:** 1 Non-Clinical Safety and Submissions, Preclinical Sciences and Translational Safety, J&J Innovative Medicine, Janssen Research & Development, Beerse, Belgium; 2 Eavise- PSI, department of Electrical Engineering ESAT, KU Leuven, Sint-Katelijne-Waver, Belgium; 3 Scientific and In vivo Strategies, Preclinical Sciences and Translational Safety, J&J Innovative Medicine, Janssen Research & Development, Beerse, Belgium; 4 Global Safety Pharmacology, Preclinical Sciences and Translational Safety, J&J Innovative Medicine, Janssen Research & Development, Beerse, Belgium

**Keywords:** animal behavior, CNS effects, computer vision, longitudinal behavioral assessment, preclinical animal models, safety pharmacology, toxicology, videomonitoring

## Abstract

Behavioral monitoring of laboratory animals is essential for evaluating drug safety, yet existing assessments are typically limited to in-room observations by technicians. Here, we introduce our versatile AI model pipeline, composed of interconnected artificial neural networks that leverage end-to-end learning based solely on video-derived appearance features of canines. This non-invasive approach enables detailed mapping of activity, behavior and clinical signs at individual animal level under diverse conditions. To validate its real-world application, we conducted extensive field testing on hours of footage. Trained on a large, annotated dataset, our model can accurately multi-track up to three group-housed canines using color-coded reflective harnesses, achieving high re-identification accuracies (≥92.5%) and IDF1 scores up to 99.9%. AI-derived locomotor activity showed a strong correlation with accelerometer-based measurements (r = 0.965). Our AI model detects 11 behavior and clinical observation classes, with a mean class accuracy of 48% and individual accuracies up to 93%. As such, a detailed time-specific quantitative output is available for activity, mobility, pose, eating, drinking and specific clinical signs (ataxia, anxiety, circling, convulsions, head shaking, involuntary muscle movements, limping, limb stiff, vomiting). Our innovative approach brings holistic behavioral and health monitoring in canines closer to routine practice and contributes towards the 3Rs principles.

## 1 Introduction

Before new medicines reach clinical trials in humans, a thorough non-clinical safety evaluation is made to assess potential safety risks and to guide safe clinical dosing strategies. Non-clinical safety assessments involve *in vivo* studies typically conducted in rodent and/or non-rodent species, providing complementary insights into potential effects of drug candidates on various physiological systems. These *in vivo* studies are guided by the 3Rs principle (reduce, refine, replace) to minimize the use and discomfort of animals. Among non-rodent species, Beagle dogs are frequently used because of their well-characterized biology and suitability for long-term observations.

In these non-clinical *in vivo* studies, the evaluation of behavioral changes and/or abnormalities (Clinical Observations or ClinObs) is crucial for understanding potential safety concerns in humans. Common clinical conditions can include altered activity level or behavior (e.g., increased anxiety or aggression), gastrointestinal signs (e.g., diarrhea or vomiting), motor deficits (e.g., limping) and neurological events (e.g., convulsion). The nature and/or severity of the observed abnormalities, with neurological signs as an important driver, heavily influences decisions to advance or halt a drug candidate. To enable correct decision making, it is critical that significant events are detected and correctly interpreted, distinguishing true drug-related effects from spontaneous occurrences. This is essential for ensuring that safety concerns are neither overlooked nor misjudged. Since neurological clinical signs have a low spontaneous incidence in many laboratory animal species, including canines (Orciani et al., 2024), quantifying the physiological baseline is extremely challenging. To increase the likelihood of event detection, both during baseline and study phases, it would be beneficial to detect premonitory signs (warning signals) which are certain behaviors that foreshadow the actual event (Authier et al., 2019). In canines, for example, convulsions are most frequently preceded by tremors and ataxia (Authier et al., 2019).

Traditionally, the presence of behavioral changes and/or ClinObs are assessed in-person by trained researchers which presents several limitations, including time constraints, subjectivity, and the effect of human interference on animal behavior. Video surveillance can eliminate these gaps through continuous monitoring of animal behavior. This can be combined with activity tracking methods (such as Actiwatch (Cools et al., 2020)) that provide a detailed overview of the animal’s activity level over time, allowing for the identification of changes that may warrant further investigation of the corresponding video footage. These manual video analysis approaches still remain time- and labor-intensive. Additionally, not every behavioral change is associated with a variation in activity level, meaning that significant events could still be overlooked.

To achieve truly continuous monitoring of animal activity and abnormal behaviors, innovative approaches are required that go beyond traditional in-room observations and wearable devices (see also (Berridge et al., 2024)). Artificial intelligence (AI)-driven video analysis, powered by computer vision, offers a promising solution by delivering a detailed time-specific and quantitative output on individual animal level. This enables the investigation of behavioral patterns and premonitory signs, and an objective quantification of spontaneous occurrences compared to on-study events. While AI-based monitoring technologies for rodents are widely explored and commercially available for several years (Isik and Unal, 2023; Kahnau et al., 2023; Sillito et al., 2025), similar solutions for non-rodent species remain undeveloped.

Overcoming the limitations of early simple animal tracking methods that were standard in behavioral pharmacology, the emergence of deep learning brought supervised methods that improved resolution and flexibility. Markerless pose estimation frameworks such as DeepLabCut (Lauer et al., 2022) and SLEAP (Pereira et al., 2022) enabled user-defined labeling of rodent poses with high precision. Most of these approaches are keypoint-based, reducing animals to skeletal landmarks (e.g., limb joints, nose, or tail base) that feed into behavior classifiers. Commercial tools like EthoVision XT (Noldus) extend this paradigm, offering interpretable pipelines for rodent ethology. However, while efficient for gross motor actions, keypoint-only representations often miss clinically important but subtle events—such as tremors, micro-movements, or fine distinctions like sniffing versus drinking—since appearance cues are discarded. Applications of keypoint tracking in non-rodent species are emerging: pose-based recognition of activity states in rhesus macaques using a 26-camera setup (Bala et al., 2020), vision-based cattle tracking for welfare monitoring (Wu et al., 2021), and camera-based detection of sow induced piglet-crushing (Gan et al., 2025). These studies illustrate feasibility across species but remain task-specific and limited in scope compared to rodent-focused systems.

To address the limitations of sparse keypoint data, recent work has shifted toward appearance-based deep learning. By learning directly from pixels, these models can capture subtle postural and textural cues that keypoints overlook. DeepEthogram (Bohnslav et al., 2021) uses a two-stream Convolutional Neural Network (CNN) for this, albeit constrained to a classic top-down camera viewpoint. In the computer vision literature, viewpoint-free methods were presented, including AnimalMotionCLIP (Zhong et al., 2025), which links visual motion with semantic descriptors, and MammalNet (Chen et al., 2023), a large-scale wildlife recognition framework. In canines, end-to-end models could even predict emotional states from still images, underscoring the potential of appearance-based AI for clinically relevant behavioral monitoring (Franzoni et al., 2024). The key advantage here is sensitivity to subtlety; the main challenge remains the need for large, diverse datasets to train these models.

In parallel, unsupervised methods such as motion-mapping (Berman et al., 2014) and hidden Markov models (Wiltschko et al., 2015) have been used to discover behavioral “syllables” without predefined labels. While powerful for exploratory ethology, these approaches are less compatible with pharmaceutical safety studies, where predefined and interpretable ClinObs categories are required for regulatory acceptance.

Together, these developments illustrate the trade-off between interpretability, annotation burden, and sensitivity. Our present work builds on this landscape by advancing appearance-based end-to-end learning tailored to non-clinical safety studies in canines, combining clinical interpretability with the capacity to detect subtle behavioral and clinical signs. We created an integrated AI model pipeline composed of different Artificial Neural Networks (ANNs) capable of monitoring individual canine activity, behavior and ClinObs; across single and group-housed conditions.

In this work, we present our unique, integrated AI model pipeline which we extensively validated in real-world scenarios involving hours of footage (>18 million frames). Thanks to our efficient annotation strategy using a single dot to mark the animal, we were able to reach an astonishing >1.8 million annotated frames. This large dataset enabled us to develop ANNs that can recognize a variety of behaviors (eating and drinking) and ClinObs (ataxia, anxiety, circling, convulsions, head shaking, involuntary muscle movements, limping, limb stiff, vomiting), purely on video data without the requirement for additional sensors or intermediate keypoint representations. Additionally, our use of non-obtrusive visual identifiers resulted in robust individual animal tracking which is crucial to generate read-outs on individual animal level. This strategy worked even in challenging group-housed settings and complex front-view multi-camera set-ups where occluders such as enrichment objects, sleeping spots and groupmates can obstruct the animals, their movement and interactions. Finally, we designed our ANNs to be generic, which allows for their application for diverse observations even across species, provided that sufficient retraining is performed.

## 2 Materials and methods

### 2.1 Animals and housing

**FIGURE 1:**
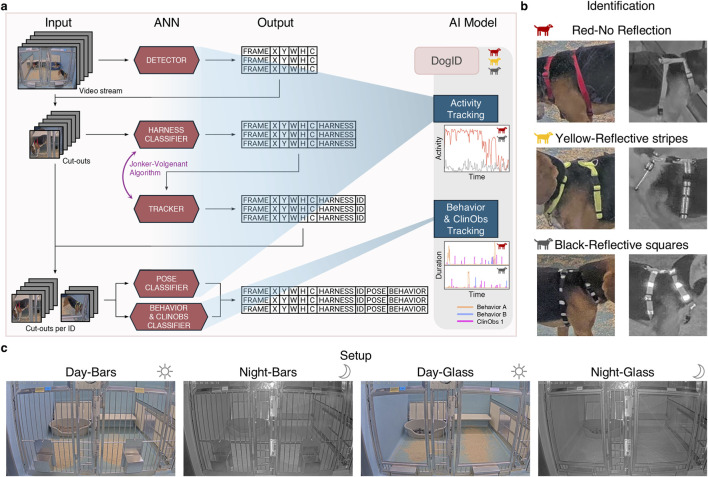
Concept for AI-based continuous monitoring of canines. **(a)** Illustration of our pipeline with interconnected ANN modules that are combined into an integrated AI model for activity, behavior and ClinObs tracking. Per ANN module, the required inputs and the generated outputs are shown. First, a detector ANN detects all canines in a video stream and subsequently generates a table with rectangular coordinates of each detection in each frame and its corresponding confidence. These are used to extract cut-outs of the individual animals in the video stream, which are then fed into a harness classifier, returning the specific harness the animal is wearing. The tracker module subsequently links detections from the same animal of different frames together in a single track. These tracks are then used to group the cut-outs separately per animal, which serve as input for the last two ANNs (pose classifier and behavior and ClinObs classifier). **(b)** Colored harnesses with reflective patterns used for animal identification and tracking: Red-None (R–N), Yellow-Reflective stripes (Y-St) and Black-Reflective squares (B-Sq). **(c)** Our two different setups (bar and glass fronts) with their respective day and night vision. To allow correct recognition of behaviors and ClinObs, front view cameras are applied which are installed at ± 1.2 m height.

Video surveillance cameras (AXIS P3235-LVE or AXIS P3245-LVE) were positioned in front view, outside of the animal bins at ±1.2 m height (Figure 1c). Data was captured at 25 or 30 frames per second (fps). All the data used to train our models was solely sourced from historical video footage of previously performed in-house studies and colony animals and originated from five experimental rooms with the same housing and camera setup. All procedures were approved by the ethics committee on Animal Experiments of the research center of Johnson and Johnson, located in Beerse, Belgium. Animals were housed in accordance with the *Guide for the Care and Use of Animals,* European Directive of 2010 (2010/63/EU) on the protection of animals used for scientific purposes, the Belgian and Flemish Region implementing legislation, and in an AAALAC-accredited facility under controlled temperature and humidity and maintained on a 12 h light/dark cycle. The studies reported here were compliant with the ARRIVE Guidelines for reporting animal research. Animals were under the care of trained biotechnicians with veterinary oversight and received appropriate veterinary care if needed. Enrichment was always provided to colony and study animals (toys, blankets, etc.) as well as sleeping space (baskets or beds).

### 2.2 Annotations

Table 1 provides an overview of the total amount of annotated data. Only for the ClinObs, the majority of the data originated from the setup with bars, as the glass fronts were only recently implemented. All data was labeled by a pool of nine people with experience (5–20 years) in canine behavior, and an additional quality control (QC) check was performed by a single expert. All ClinObs videos were approved as being representative prior to labeling by three experts as previously described (Eberhardt et al., 2025).

**TABLE 1:** Overview of the manually annotated video data for each ANN. Per ANN and per relevant subsection (different setup, dataset, or class): number of annotated video frames and annotation type (bounding box or dot).

ANN	ANN subsection	N° frames		Annotation type
		Day	Night	
Detector (balanced bars/Glass)	Bars	196002	103300	Bounding box
	Glass	65176	8937	
Harness classifier (balanced bars/Glass)	*Initial dataset* Red-none Yellow-stripes Black-squares Black-dots Grey-fluo No harness	*381* 70 62 64 60 61 64	*385* 65 66 62 65 60 67	Dot
	*Final dataset* Red-none Yellow-stripes Black-squares	*18004* 6878 6042 5084	*17252* 5268 6260 5724	
Tracker (bars)	Day-S	2878	​	Dot
	Day-C	1746	​	
	Night-S	​	2301	
	Night-C	​	1713	
Pose classifier (bars)	Lying	10632	2270	Bounding box
	Sitting	4663	642	
	Standing	23625	5093	
	Standing up	4892	345	
	Standing down	293	40	
Behavior classifier (balanced bars/Glass)	Eating-hopper	45250	​	Bounding box/Dot
	Eating-bowl	35867	​	
	Drinking	60804	16942	
	None	258020	54014	
Behavior and ClinObs classifier (majority only bars)	Eating-hopper	51148	​	Bounding box/Dot
	Eating-bowl	35867	​	
	Drinking	57224	16942	
	Anxiety	8762	​	
	Ataxia	39878	3116	
	Circling	13021	3059	
	Convulsion	24098	3059	
	Head shaking	3816	2016	
	Inv. Muscle movements	72526	16162	
	Limb stiff	3817	86	
	Limping	47334	11131	
	Vomiting/Retching	7608	3427	
	None	696844	190825	

We deliberately included challenging data in our datasets to better reflect real-world conditions. These difficult cases comprised examples where distinguishing between similar behaviors or clinical observations proved particularly difficult–for example, sniffing the drinking nipple (*not drinking*) versus actual drinking. Other challenging examples included occluded or overlapping animals, and observations that were very similar, such as rolling versus convulsion. Adding this complexity during training enhanced model robustness; and during evaluation allowed for more rigorous assessment.

All annotations were performed using an in-house customized version of the open-source Computer Vision Annotation Tool (CVAT) (https://github.com/cvat-ai/cvat). We started with bounding box annotations which we replaced by a dot-2-box strategy to decrease labeling time once our detector ANN was finalized. In the latter, a single center-point (dot) was placed on each animal which was matched with the center point of a predicted bounding box by our trained detector. The latter was subsequently used to generate cut-outs for ANN training, validation and testing.

### 2.3 Performance metrics

Model performance was assessed using accuracy (top-1), precision, recall, and the derived F1 score, all obtained from confusion matrices. Accuracy refers to the proportion of correctly classified frames. However, this measure can be inflated by the presence of class imbalance, as canines spend much of their time in the dominant background class *none* and spend most of their time lying or standing. To address this, we report both top-1 accuracy (micro accuracy across all frames) and class accuracy (the mean of individual class accuracies). Precision quantifies the proportion of predicted positive frames that are correct, while recall measures the proportion of true positive frames identified. Their harmonic mean, the F1 score, provides a balanced measure of both. Accuracy and F1 were calculated at the threshold, maximizing the F1 point on the precision–recall curve.

Tracking performance was measured using standard metrics (Ristani et al., 2016). Multi Object Tracking Accuracy (MOTA) captures overall error by combining missed detections, false positives, and identity switches. To evaluate tracking consistency, we used IDR, IDP and IDF1: identity recall (IDR) quantifies the fraction of the ground-truth tracks that are correctly recovered, identity precision (IDP) measures the proportion of predicted tracks that match ground-truth tracks, and IDF1 is their harmonic mean.

### 2.4 ANN modules composing our AI model pipeline

All ANN training, validation and testing were performed on the Domino platform (https://domino.ai/platform) using a single Nvidia L4 GPU (24 GB of memory).

#### 2.4.1 Detector

The data (Table 1) was split into train-validation-test sets according to a 70%-10%–20% ratio, and per video rather than on a frame-level. Since frames from the same video likely resemble each other, they should not be distributed across different subsets, as this could hinder the ability to determine whether the detector is overfitting. Additionally, we balanced day/night, bars/glass and single/multiple canines across the different subsets.

We compared a variety of object detectors for which we selected the best architecture and training parameters on the training and validation datasets: YOLOv2 (Redmon and Farhadi, 2017), YOLOv3 (Redmon and Farhadi, 2018) YOLOv4 (Bochkovskiy et al., 2020), YOLT (Van Etten, 2018), D-YOLO (Acatay et al., 2018) and ResNet-YOLO (Ophoff et al., 2022).

Our most performant detector YOLOv2 trained with the Complete Intersection-over-Union (CIoU) loss introduced in (Bochkovskiy et al., 2020) (Results 3.3.1), was fine-tuned from ImageNet pretraining and optimized with stochastic gradient descent. A cyclic learning rate was used ranging from 2 × 10^−8^ to 10^–3^. The training was conducted with an effective batch size of 32 over a maximum of 30 epochs. We selected the best model on the validation set after training. The loss function employed the Complete Intersection-over-Union (CIoU). The input images were resized to 640 × 384 pixels. The input data was normalized using ImageNet statistics and augmentation included random horizontal flipping, color jitter and geometric jitter.

#### 2.4.2 Animal identification

##### 2.4.2.1 Fur approach

We initially investigated fur patterns for animal identification, training a ResNet-18 model (He et al., 2016) a dataset of 40 canines (50 images per canine) with 5-fold cross-validation and data augmentation. This approach was validated using a nearest-neighbor classifier and three-crop evaluation, this resulted in a 63.7% top-1 accuracy, which proved insufficient for reliable tracking (data not shown). The suboptimal performance was likely due to the smaller dataset combined with the variability in fur patterns depending on which part of the animal was visible. As accurate tracking of individual animals is essential, we opted to use the harness approach for identification.

##### 2.4.2.2 Harness approach

To overcome the limitations of fur-based identification, we shifted to using harnesses with distinct visual features. Each harness had a unique daytime color and a reflective pattern visible at night. As the maximum number of individuals in a group is typically three, we only needed three distinct IDs. For larger group sizes, animals can be housed in sub-groups of ≤3 animals each. To identify the most distinguishable combinations, we started with six harness types: *Black-Dots, Black-Reflective squares, Grey-Fluo, Yellow-Reflective stripes*, *Red-No reflection* and *No harness.*

All harness classifier models consist of a ResNet-18 (He et al., 2016) backbone trained with a batch size of 100 and cross-entropy loss. The 6-ID and 4-ID models were finetuned from ImageNet1k weights using SGD with a learning rate of 0.01 for 50 epochs. The 3-ID model was finetuned from the 4-ID model using a slightly lower learning rate of 0.008 and trained for 20 epochs. Input images were resized, normalized to ImageNet statistics, and augmented with random horizontal and vertical flipping. Each model used 10-fold cross-validation to select optimal hyperparameters.

- 6-ID Model: Trained on the initial dataset (Table 1) with all six harness types. After hyperparameter tuning, the model was trained on the full training set and evaluated on the test set, achieving a top-1 accuracy of 94.2%. Based on these results, we dropped the underperforming harnesses *Black-Dots* and *Grey-Fluo* (see Supplementary Figure S1).
- 4-ID Model: Retrained on the reduced set of four harnesses: *Black-Reflective squares*, *Red-No reflection*, *Yellow-Reflective stripes*, and *No harness.* This model achieved a top-1 accuracy of 99.0%. As performance was consistent throughout all classes, we chose to drop the *No harness* class to ensure all canines were treated equally during a study.
- 3-ID Model: The final model was trained on three harnesses: *Black-Reflective squares* (B-Sq), *Red-No Reflection* (R-N), and *Yellow-Reflective stripes* (Y-St). To improve robustness, we expanded the dataset to include more challenging scenarios, such as occlusions and cut-outs containing multiple harnesses (Table 1). This model was evaluated on a separate set of videos, which also served as the evaluation set for the tracker (Results 3.3.3).

#### 2.4.3 Animal tracking and reID

**FIGURE 2:**
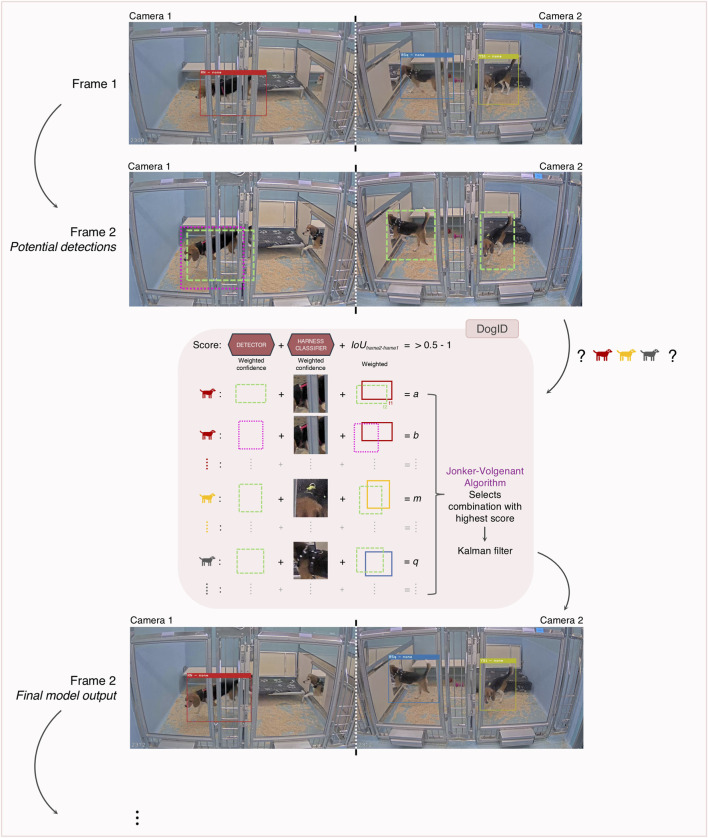
Multi-camera processing for accurate tracking and reID. Illustration of our tracking and reID strategy in group-housed animals across multiple camera views. All potential detections within a frame (across multiple camera views) are processed by the reID model (red shaded box) that utilizes the weighted confidence scores from both the detector and the harness classifier model to select the optimal combinations. Furthermore, if a canine with the same ID was detected in the previous frame, we employ the Intersection over Union (IoU) metric to further refine the bounding box selection. Using these three parameters, a score is calculated for each potential detection in the frame, with a higher score indicating greater certainty in our models' predictions. Finally, we apply a modified Jonker-Volgenant algorithm to find the combination that yields the highest overall score. In a last step, we use a Kalman filter for tracking the bounding boxes over time, smoothing out abrupt changes and filling in missed detections.

To optimally select the correct detections and assign the correct animal ID, we developed the following strategy. The detector renders several potential detections within a frame (or across multiple camera views) which are all processed by the harness classifier model. To select the optimal combination, we utilized the confidence scores from both the detector and the harness classifier. If a canine with the same ID was detected in the previous frame, we included the IoU score between the bounding box in the previous and current frame to further refine the selection of bounding boxes (Figure 2). Using this information, we calculated a score for each potential detection in the frame using the following formula:

$$score=\frac{\lambda_{d}C_{d}+\lambda_{r}C_{r}+\lambda_{IoU}IoU}{\lambda_{d}+\lambda_{r}+\lambda_{IoU}}$$

$C_{d}$ and $C_{r}$ represent the confidence scores of the detector and Harness classifier model, respectively, and range from 0 to 1. IoU is the score between the current bounding box and the bounding box in the previous frame. When there is no previous detection, this score is set to 0. $\lambda_{d}$ , $\lambda_{r}$ and $\lambda_{IoU}$ are weighing factors for the corresponding scores. We achieved the best results with respective values of 1.0, 0.75, and 1.0.

In this way, each detection is given a score between 0 and 1 for every ID, with a higher score indicating greater certainty in our models' predictions. To filter out less certain detections and reduce the likelihood of false positives, only detection/ID combinations with a score >0.5 were retained. We subsequently used a modified Jonker-Volgenant algorithm (Jonker and Volgenant, 1987; SciPy, 2008; Crouse, 2016) to find the combination that yields the highest overall score. A significant advantage of this algorithm is that it prevents the same ID from being detected twice. In addition, because the algorithm runs on every frame, a misclassification on a certain frame does not influence the misclassification probability in subsequent frames.

As camera views of adjacent kennels are processed together, the same approach was applied in group-housed conditions when animals were able to move across different camera views (Figure 2). The Harness classifier model processes every detection across all different camera views, and the modified Jonker-Volgenant algorithm continues to select the best combination, thus preventing duplicate IDs across the various camera views. Another advantage of this simultaneous processing is that the algorithm can compare all detections and assign IDs based on its certainty. For example, if only two of three harnesses are clearly visible, the algorithm can correctly assign those two IDs and infer the ID of the third animal, even though the harness itself is not visible.

Finally, we used a Kalman filter (Kalman, 1960) to track the bounding boxes over time, smooth out abrupt changes and fill in missed detections.

We evaluated the performance of the tracking module on a collection of video snippets covering day and night with animals either mostly separate (Day/Night-S) or clustered (closely together with overlapping bounding boxes, Day/Night-C) (Table 1).

#### 2.4.4 AI activity tracking and mobile/immobile

For the AI activity tracking, in postprocessing, the Euclidean distance (in pixel) was determined between the center of the current bounding box and the center of the bounding box on the previous frame/of the previous detection (Figure 4a). In this way, a measure of movement was obtained for every animal on every frame. These movements were then binned per time frame (generally per minute) to obtain data for visualization in “actigrams” that provide a detailed plot of activity levels over time per animal.

To get a more general overview of the animals’ activity, the movements described above were classified on a frame-level as ‘mobile’ or ‘immobile’ based on a cutoff (Figure 4a, and Methods 2.5.2). Mobile refers to animals moving from one point in space to another, while immobile refers to animals remaining in the same location.

#### 2.4.5 Pose classifier

We considered five poses: *Lying*, *Sitting*, *Standing*, *Standing Up* (on hind legs) and *Standing Down* (on front legs, e.g., when jumping off the bench). The initial dataset was split with similar ratios of the different poses in the subsets.

ResNet-18 (He et al., 2016) served as a backbone for the pose classifier, trained using the cross-entropy loss for up to 50 epochs using a batch size of 64. Again, the best model was selected based on its performance on the validation set. SGD and a cyclic learning rate (varying between 1 × 10^−3^ and 2 × 10^−2^) were used for optimization. The inputs were letterboxed (using gray to pad the borders) and resized to 256 × 256 pixels, normalized, augmented with random horizontal flipping, color jitter. We used class balancing during training, as well as detector-generated bounding boxes to make the model robust to imperfect bounding boxes.

#### 2.4.6 Behavior classifier

The behavior classifier receives sequential cut-outs of a single canine over time from the tracker module. Unlike previous models, which primarily process spatial information, this model also incorporates temporal information by analyzing sequential frames. This additional temporal context is crucial, as behaviors are often not apparent from a single frame alone.

We considered three behaviors: eating, drinking and *none* (no behavior of interest) (Table 1). As animals can be provided with food from either a hopper or a bowl, video material from both conditions was subdivided into respective subclasses. This subdivision into more homogeneous classes increased accuracy. During postprocessing, both subclasses were recombined into a single *eating* category. Additionally, we included several challenging *not drinking* and *not eating* fragments in which animals were standing near or sniffing the drinking nipple or food hopper/bowl without actually drinking or eating. This was done to help the model learn to distinguish between similar appearances more accurately.

The data was split into “training-validation-test” according to a 60%-15%–25% ratio with balanced day/night and bars/glass. Since the behavior model operates on entire videos and individual videos cannot be divided across different data sets, these ratios were approximate at the frame level. To address the class imbalance in the dataset during training, we employed random weighted sampling.

We fine-tuned a small Vision Transformer (ViT-S/16) with joint space-time attention (Dosovitskiy et al., 2020; Arnab et al., 2021; Tong et al., 2022), initialized from a VideoMAE checkpoint, pretrained on Kinetics-400(Tong et al., 2022), as our behavior classifier. Inputs were 16-frame clips at 224 × 224, sampled with a temporal stride of 4, yielding a 64-frame window (∼2.5 s at 25fps). Training used a AdamW optimizer with a cosine decay learning rate schedule (base 1 × 10^−3^, minimum 1 × 10^−6^), weight decay of 0.05 and 3 warm-up epochs, for a total of 15 epochs. Similarly, the best model on the validation set was selected. Gradient accumulation yielded an effective batch size of 150. Clips were letterbox-padded and normalized; augmentations included random horizontal flipping and temporal jitter of the bounding boxes to simulate detector noise. To further mitigate imperfect bounding boxes, we added 50 pixels of padding to each side of the bounding box to make sure the entire animal was visible.

#### 2.4.7 ClinObs classifier

The ClinObs classifier is an extended version of the behavior classifier. Consistent with the approach used for the behavior model, this expanded dataset also included examples designed to represent nuanced clinical observations and the challenges in distinguishing them from similar behaviors and background activity (Table 1).

Building upon the behavior classifier, we trained the ClinObs model using the same ViT-S/16 architecture with joint space-time attention (Dosovitskiy et al., 2020; Arnab et al., 2021; Tong et al., 2022), initialized from a VideoMAE checkpoint pretrained on Kinetics-400 (Tong et al., 2022). We split the data into training, validation, and test sets using the same 60%-15%–25% ratio, still maintaining balanced representation of day/night conditions and bars/glass environments. We used a batch size of 14 with gradient accumulation over 10 batches, yielding an effective batch size of 140. The model’s input was the same as the behavior model: 224 × 224 images, with each input clip consisting of 16 frames sampled with a stride of 4. We still address the class imbalance with random weighted sampling. We trained the model for 75 epochs, including a 15-epoch warm-up period, utilizing an AdamW optimizer with a cosine decay learning rate schedule (base 1 × 10^−3^, minimum 1 × 10^−6^ and weight decay of 0.05). Once more, the best model on the validation set was selected. Consistent with the behavior model, we applied letterbox padding and normalization of the clips, implemented temporal jitter augmentation, and added 50 pixels of padding to each side of the bounding box. To prevent overfitting, we implemented mixup (Zhang et al., 2017) and cutmix (Yun et al., 2019) augmentation techniques, alongside RandAug (Cubuk et al., 2020) with 9 augmentations and a magnitude of 15. As before, we split eating and eating-bowl during training; additionally, we split ataxia and IVM into subcategories representing varying degrees of severity (slight-moderate-severe) during training, with these subcategories merged for testing.

### 2.5 Field validation of our AI model pipeline

For all field validations explained below, cameras recorded at 25 or 30fps and a subsampling of 4 was applied in our AI pipeline; meaning that only every fourth frame was analyzed. We applied this subsampling to maintain an acceptable, close-to-real-time processing speed of our pipeline which is important for future implementation. The performance of our pipeline was assessed by repeatedly processing a 1-h single-camera video and a 1-h double-camera video ten times each. The average processing time for the single-camera feed (30fps) was 54 min and 0 s (SD 32 s), demonstrating real-time performance. In contrast, processing the double-camera feed (30fps) required an average of 1 h 48 min and 58 s (SD 102 s). All tests were conducted on the same single Nvidia L4 GPU.

Table 2 provides an overview of the different video fragments that were analyzed for each field validation.

**TABLE 2:** Overview of the video fragments analyzed for each field validation. Number of selected video fragments per day/night, including snippet duration, total video time and number of frames that were analyzed. It is specified whether the fragments contain single- or group-housed animals, the type of enclosure (bars or glass fronts), how the ground truth was established and whether the animals were wearing harnesses.

Field validation	N° video fragments		Length video fragment	Total video time	Total N° frames	Single/group housed	Glass/Bars	Ground truth	Animals wearing harnesses?
	Day	Night							
AI activity tracking									
Activity tracking	6	6	2 h	24 h	N.A.	Group (N = 3)	Bars	Actiwatch-nano®	Yes
reID and tracking	11	10	5 min	101.5 min	39,748	Group (N = 3)	Bars	Manual	Yes
	7	7	5 min	70 min	26,225	Group (N = 3)	Glass	Manual	Yes
	9 (3/group)	9 (3/group)	5 min	90 min (30 min/group)	33,939 (∼11,300/group)	Group (N = 2)	Bars	Manual	Yes
Mobile/immobile	14	0	10 min	140 min	50,713	Single	Bars	Manual	No
	3 *	3 *	5 min	90 min	28,771	Group (N = 3)	Bars	Manual	Yes
	* Each video fragment contained 3 different animals for which the mobile/immobile was assessed individually								
Pose	14	0	10 min	140 min	50,713	Single	Bars	Manual	No
Behaviors									
Eating-hopper (incl. “not eating”)	4	0	15 min	1 h	22,511	Single	Bars	Manual	Yes
	4	0	15 min	1 h	22,518	Single	Glass	Manual	Yes
Eating-bowl (incl. “not eating”)	4	0	15 min	1 h	22,201	Single	Bars	Manual	Yes
	4	0	15 min	1 h	22,170	Single	Glass	Manual	Yes
Drinking	13	2	Variable	7.9 min	2,969	Mixed	Bars	Manual	Yes
	12	2	Variable	9.5 min	3,579	Mixed	Glass	Manual	Yes
“Not drinking”	7	2	Variable	6.2 min	2,339	Mixed	Bars	Manual	Yes
	12	2	Variable	7.2 min	2,702	Mixed	Glass	Manual	Yes
Real-life validation	10	0	4 h	40 h	∼1,000,000	Single	Bars	Manual (event-level)	Yes
ClinObs									
Ataxia, IVM Head shaking	27 (9 animals, 3 days)		24 h	648 h	∼17,500,000	Mixed	Bars	*In person* and Manual event-level	Yes

#### 2.5.1 Field validation of activity tracking, reID and behavioral classification

We selected six female Beagle dogs from our colony to wear the three different harness-types: Y-St (two animals), B-Sq (two animals) and R-N (two animals). Animals were group-housed with three groups of N = 2 in a room with bars to include all three possible harness combinations. Subsequently, groups were reorganized into two groups of N = 3 in a room with glass fronts, followed by bars. After a habituation period in a room with bars, all animals (two groups of N = 3) were equipped with an Actiwatch-Nano® accelerometer attached to their harnesses to record their activity.

For the activity tracking validation, 2-h videos were selected with varying activity levels in all animals, both during day and night (Table 2). The Actiwatch-Nano® provided one read out (activity count) per minute. These were compared to the AI activity levels which were calculated using the Euclidean distance (in pixel, as explained above in “AI activity tracking and mobile/immobile” and Figure 4A). Accelerometer and AI data were compared in two ways: i) by visual inspection of individual actigrams; and ii) by assessing their correlation. For the latter, we calculated the non-parametric Spearman correlation coefficient using Graph Pad Prism 10.1.2 for activity read-outs per minute, and binned per 15 min and per 2-h period.

To validate our tracking and reID model, snippets were specifically selected to include a variety of interactions in group-housed animals to challenge our model: walking or running around, animals crossing each other, playing, resting separately and together, etc. (Table 2). The ground truth ID was manually established on every frame and tracking metrics were calculated using the motmetrics python library (Heindl, 2019).

For the validation of the behavioral classifier, we evaluated the performance of *drinking*, *eating* from a food hopper, *eating* from a bowl, and *none* (Table 2). The *none* category was evaluated on the *eating* and *drinking* fragments as they contained typical *none* behavior like animals walking around, jumping on and off bench, animal-animal interactions, etc. To challenge our model, we also included several *not eating* and *not drinking* fragments. The overall confusion matrix was generated by summing frames across all videos for both the manually established ground truth and AI model.

In a final real-life validation, 4-h footage spanning the entire food access period, was analyzed for 10 animals (Table 2). Our model predictions were manually checked on video and the entire footage was also visually checked for missed detections by the model. Accuracy was calculated on event-level:

$$\frac{\#\;events\;correctly\;detected}{total\;\#\;predicted\;events+missed\;events}\;x\;100$$

#### 2.5.2 Field validation of mobile/immobile

The validation of mobile/immobile was done in two parts (Table 2).

For the first part, 10 min fragments were selected of single-housed animals from prior studies in which either increased or decreased activity was registered. The ground truth whether the animal was mobile or immobile was manually established on every frame. To determine the most optimal cutoff for mobile/immobile classification, an ROC (Receiver Operating Characteristic) curve was calculated that plots the TPR (True Positive Rate) in function of the FPR (False Positive Rate) for every cutoff. We evaluated values between 0 and 20 with 0.01 increments and calculated the Youden’s Index for every point to find the best trade-off between TPR and FPR:

$$Youden^{\prime }s\;Index=sensitivity+specificity-1=TPR-FPR$$

The highest Youden’s Index of 0.91 corresponded to a cutoff of 10.0. This cutoff was subsequently validated in group-housed animals.

#### 2.5.3 Field validation of pose classification

The same 10 min video fragments with increased/decreased activity were analyzed to have a more balanced distribution of the more “excited” poses (standing, standing up and standing down) vs. the more ‘quiet’ poses (sitting and lying) (Table 2).

#### 2.5.4 Field validation of ClinObs classification

We selected nine female Beagle dogs and randomly divided them into three groups of three animals wearing the different harness types. All animals were observed for two baseline days prior to dosing. On the third day, each group received a different treatment by oral gavage: 1) Acepromazine 2.5 mg eq/kg (Sigma-Aldrich®, Burlington, MA, US); 2) Transcutol® HP 1500 mg/kg (Gattefossé, Saint-Priest, France); and 3) Water as vehicle control (Figure 7a). As per standard protocol, we observed the animals *in person* for any change in behavior and/or ClinObs during specific time periods post-dosing: 5 min, 30 min, 1 h, 2 h, 4 h, 7 h, and 10 h. To assess whether our AI model was able to detect the same ClinObs and behavioral changes as noted during the *in person* observations, we analyzed the 24-h footage of both baseline days and the dosing day using our AI pipeline, followed by human QC of significant/aberrant AI signals (as illustrated in Figures 7c,d). For visualization purposes, framewise AI predictions were summarized per minute by binning the number of predictions per class within that minute.

On top of the framewise predictions, we implemented an objective, adaptive baseline thresholding strategy to highlight significant differences between the baseline and treatment days. For this, a moving average with a 5-min time window was applied on the framewise predictions to account for the temporal aspect. Individual thresholds were then calculated for each animal and each ClinObs class by determining the *minimum* number of predictions per minute required to suppress all predictions for that class during the baseline period. This threshold was subsequently applied to the dosing day data.

## 3 Results

### 3.1 AI model approach

Our goal was to develop an integrated AI model for continuous monitoring of canine activity, behavior and clinical observations (ClinObs) tailored for experimental purposes in nonclinical research and compliant with animal welfare standards. To accomplish this, we developed a pipeline consisting of multiple ANNs (Figure 1a): a detector, an identification and tracking module, followed by pose, behavior and ClinObs classifiers. As our AI model would be implemented during both day and night, animals are wearing colored harnesses with distinct reflective patterns (Figure 1b) to facilitate visual identification. In addition, two distinctive animal room setups were used, with either bar or glass fronts (Figure 1c). Unlike typical rodent setups that utilize top-down camera views, we apply front view cameras for accurate recognition of behaviors and ClinObs.

In our pipeline, the interconnected ANNs rely on the output of the previous models, making it crucial to develop robust ANNs and add redundancy to our pipeline. On top of a performant detector ANN, the most crucial part in our integrated AI model is accurate animal identification and tracking as the ANNs in subsequent stages of the pipeline require consistent cut-outs of the same animal through time for correct predictions. In practical situations, animals are typically group-housed and have access to multiple kennels, resulting in movement across different camera views. To achieve the most performant identification and prevent duplicate IDs, camera streams of adjacent kennels are processed jointly (Figure 2 and Methods 2.4.3).

**FIGURE 3:**
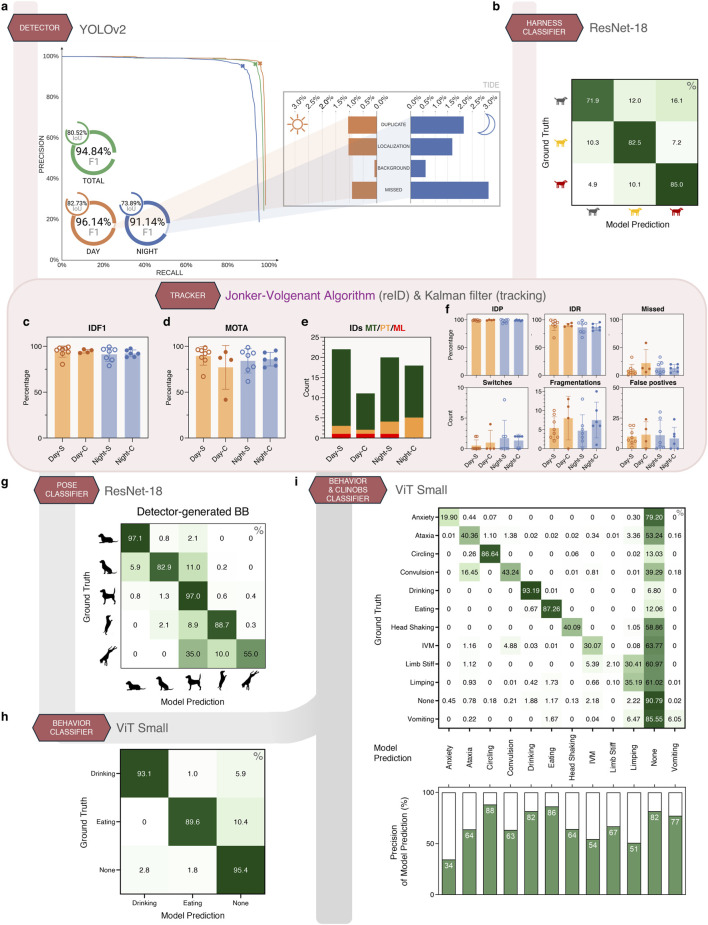
ANN Performance on test datasets. **(a)** The precision-recall curve of our highest performing detector ANN at different confidence thresholds for all (green), day-only (orange), night-only (blue) detections. The selected threshold (marked as X) of 45.6% corresponds to the optimal F1 point of the precision and recall. The TIDE analysis shows the types of mistakes made by the detector. **(b)** Performance of our harness classifier ANN. **(c–f)** Tracking performance based on the tracking and reID strategy from Figure 2. Tracking metrics are evaluated on a total of 8,638 frames giving 23,283 targets divided over several day and night videos with animals mostly separate (Day/Night-S) or clustered close together (Day/Night-C). All metrics are represented as mean ± SD with each dot representing an individual video. IDs MT/PT/ML: number of IDs that are Mostly Tracked (>80%, green)/Partially Tracked (20%–80%, orange)/Mostly Lost (<20%, red). **(g)** Performance of our pose classifier ANN using detector-generated cut-outs. False positive and false negative detections are excluded from the confusion matrix, as animals were not wearing harnesses for correct identification and tracking. **(h)** Frame-level performance of our behavior classifier ANN on manually annotated videos using dot-2-box. **(i)** Frame-level performance of our behavior and ClinObs classifier ANN on manually annotated videos using dot-2-box: recall is depicted in the confusion matrix and precision in the bar chart. IVM: involuntary muscle movement (tremors and/or twitches).

We first validated the performances of individual ANNs on their respective test sets (Figure 3). These datasets, however, do not account for error propagation across the integrated pipeline, where mistakes from one ANN may cascade to subsequent stages. To address this, we additionally performed a large-scale field validation on extended video recordings, evaluating the end-to-end performance of activity tracking (including reID and pose classification), behavior, and ClinObs classification.

### 3.2 Datasets

Our dataset consists of >1.8 million manually annotated frames across 1,125 video snippets that span a large variation of single- and group-housed canines with and without visual identifiers, different poses and especially behaviors and ClinObs seen from our fixed video surveillance cameras (Table 1). The dataset includes footage from five different animal rooms with an identical camera view and blue background, but with variables such as enrichment (toys, blankets), bedding and different front door views.

### 3.3 ANN performance on their respective test sets

#### 3.3.1 Detector

Out of the several detector ANNs evaluated (Supplementary Figure S1A), the best performing model was YOLOv2 (Redmon and Farhadi, 2017). Our detector ANN reached both high precision and recall, reflected by a F1 score of 94.8% (Figure 3a). Performance was slightly better on daytime footage (F1 of 96.1%) than during night (91.1%). This was expected given that approximately 65% of our data contained day footage, while nocturnal conditions pose greater challenges. To verify the accuracy of the generated bounding boxes that downstream ANNs rely on, we calculated the average Intersection over Union (IoU) between the detector output and the annotated bounding boxes. Our detector ANN reached an overall average IoU of 80.5%, with a lower IoU at night (73.9%) compared to day (82.7%) due to reduced visibility in greyscale imaging and the natural tendency of canines to cluster together while sleeping. These values are considered very high since an IoU ≥50% is typically regarded as a true positive match in computer vision literature (Everingham et al., 2010; Redmon and Farhadi, 2017).

A TIDE (Toolkit for Identifying Detection and segmentation Errors) analysis (Bolya et al., 2020) offered insight in the sources of errors our detector model made. It showed that all types of mistakes made by our detector ANN occurred more during the night (≤3%) compared to the day (≤1%), correlating to the ANN’s decreased performance (Figure 3a). Most errors at night were missed detections followed by duplicate detections (canines were generally not wearing harnesses in this footage) and localization errors (low IoU overlap). The detector ANN showed very low background detections (erroneous identification of non-existing canines) across both conditions, indicating a low propensity for false positives.

Note that YOLOv2 was relevant at the time of our research. Although, in the meantime novel architectures are available, we opted not to test them as our detector model and the resulting animal tracking (Section 3.3.3, 3.4.2.) are very performant and the potential gain would be minimal.

#### 3.3.2 Animal identification

Before collecting a large dataset to train a robust animal identification model, we initially gathered smaller datasets to determine the most effective approach (see Methods 2.4.2, Supplementary Figure S1B). This preliminary step allowed us to evaluate different strategies and select the best-performing methods for our final model.

Our final approach utilizes an ResNet-18-based ANN that can classify 3 IDs based on color during the day and the reflective pattern during the night: *Black-Reflective squares* (B-Sq), *Red-No Reflection* (R-N), and *Yellow-Reflective stripes* (Y-St) (Figure 1b). This ANN was trained on a larger dataset that included more challenging data, such as occluded animals and image cut-outs that contained multiple harnesses (Table 1). Our final harness classifier ANN achieved an overall top-1 accuracy of 80.3% with recognition of the different harness types varying from 71.9% (B-Sq) to 85.0% (R-N) (Figure 3B).

#### 3.3.3 Animal tracking and reID

Our strategy of combining a modified Jonker Volgenant algorithm (Jonker and Volgenant, 1987; SciPy, 2008; Crouse, 2016) with a Kalman filter across multiple camera views (Figure 2) resulted in excellent tracking and reID performance (Figures 3c–f), where reID (re-identification) is defined as assigning the correct harness color (ID) to every animal on every frame. In general, there were no notable differences in tracking performance between separate and clustered animals, apart from a tendency of more fragmentations in the clustered videos. This was also the case for day and night, where day scored only marginally higher for IDF1, IDP and IDR. Across all conditions, we reached average scores of 85.1% MOTA, 93.0% IDF1, 98.4% IDP and 88.4% IDR; and 11.0% missed detections (Figures 3c–f).

IDF1 scores for both separate and clustered animals were outstanding for day (∼94%) and night (∼91–92%) conditions, implicating that the correct ID was assigned to nearly each detected animal (Figure 3c). This was also reflected in the close to perfect IDP score (Figure 3f). Of the 71 ground truth identities, 57 IDs were mostly tracked (tracked for more than 80% of their lifespan), 11 were partially tracked (between 20%–80%), and only 3 were mostly lost (less than 20%) (Figure 3e). The MOTA score accounts for missed detections, false positives, and ID switches, which typically leads to lower values than IDF1 that focuses more on identity preservation. Nevertheless, our MOTA scores of 77.0%–89.5% still indicated strong tracking performance (Figure 3d). Indeed, apart from a few outliers, the number of missed detections was low across all video snippets, which is also mirrored in high IDR scores (∼86–90%) (Figure 3f). Considering the huge number of 23,283 targets (i.e., individual animal cut-outs), we observed a negligible number of switches, fragmentations and false positives (Figure 3f).

The fact that our IDR score was higher than the performance of our harness classifier model alone, implied that the Jonker-Volgenant algorithm had a positive effect on ID consistency.

#### 3.3.4 Pose classifier

The pose classifier receives cut-outs from a single canine through time and predicts the pose on a frame level (Figure 1a). Our ResNet-18 model reached a top-1 accuracy of 95.2% when using detector-generated cut-outs instead of manually annotated ones (Figure 3d). Lying and Standing classes were the most performant (accuracies of ∼97%), followed by Standing Up (88.7%) and Sitting (82.9%). Standing Down was the least performant class with 55.0% accuracy (Figure 3d), also related to the very low number of occurrences in our dataset compared to the other poses (Table 1).

#### 3.3.5 Behavior classifier

Our initial ViT Small model for behavior was trained for drinking and eating (Figure 3e). The model achieved a top-1 accuracy of 94.0% and a class accuracy of 92.7% calculated on a frame-level. It performed well on both behavior classes, with accuracies of 93.1% for drinking and 89.6% for eating, while rarely confusing these (Figure 3e). When errors occurred, they were typically misclassifications between a behavior and the background class (*none*), or *vice versa*, suggesting that the model has learned to distinguish these behavioral patterns. Furthermore, high accuracy on the background class *none* (95.4%) indicates a low number of false positives (Figure 3e).

#### 3.3.6 ClinObs classifier

Building onwards from the ViT Small Behavior model, our ClinObs model demonstrated promising results in detecting 11 distinct behaviors and clinical observations on a frame-level, achieving a top-1 accuracy of 79.0% and a class accuracy of 47.9%. Our model maintained strong performance on behavioral classes such as drinking and eating, highlighting its ability to reliably detect common observations. As expected, classes with limited representation in the training data such as anxiety, limb stiffness, and vomiting presented greater challenges for the model. Because of this extreme—but unavoidable—training data’s class imbalance, the performance ranged substantially between classes: drinking (93.2%), none (90.8%), eating (87.3%), circling (86.6%), convulsion (43.2%), ataxia (40.4%), head shaking (40.1%), limping (35.2%), involuntary muscle movements (IVM, 30.1%), anxiety (20.0%), vomiting (6.1%) and limb stiff (2.1%) (Figure 3f). Notably, our model occasionally confused IVM with convulsion (4.9%), convulsion with ataxia (16.6%), and limb stiff with limping (30.4%), which is understandable due to the visual similarity of these ClinObs (Figure 3f). Because of the inherent class imbalance, the model showed a greater inclination toward predicting the background class None. Across nearly all classes, the precision was higher than the recall, indicating that the model leans more towards correctly predicting instances, at the expense of missing some true positive events.

### 3.4 Field validation of our integrated AI pipeline

#### 3.4.1 Field validation of activity tracking

**FIGURE 4:**
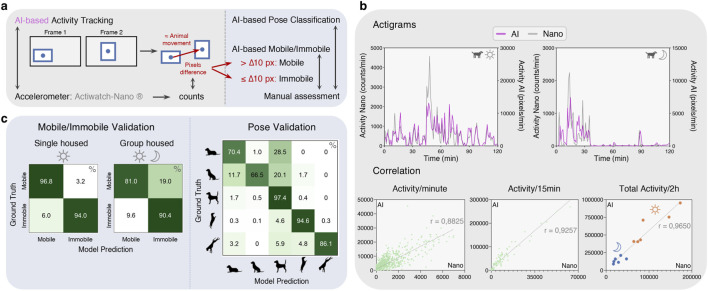
Field validation of Activity Tracking and Pose Classification. **(a)** For the AI activity tracking, in postprocessing, the Euclidean distance (in pixel) is calculated between the center of the bounding box on the current and the previous frame/detection for the same animal. The pixel difference represents a measure of movement per frame. Based on the pixel difference, a cutoff of 10 was established to classify bounding box/animal movements on a frame-level as ‘mobile’ or ‘immobile’ which are compared to manual assessment. Mobile refers to animals moving from one point in space to another, while immobile refers to animals remaining in the same location. **(b)** Comparison of AI activity tracking to Actiwatch-Nano® on individual actigram level and by correlation of activity read-outs binned per minute, 15 min and per 2 h. (**(c)**, left) Confusion matrices for mobile/immobile validation on single- (day) and group-housed (day and night) animals. (**(c)**, right) Confusion matrix for the validation of our pose classifier compared to manual ground truth on single-housed animals with increased and decreased activity.

Accurate AI Activity tracking relies on correct outputs from the detector, harness classifier and tracker (Figure 1a). To validate our AI Activity tracking, we compared it to Actiwatch-Nano®, an accelerometer-based device routinely used during nonclinical studies performed in this facility. We calculated the animals’ activity based on the movement of their bounding boxes (Methods 2.4.4, Figure 4a).

**FIGURE 5:**
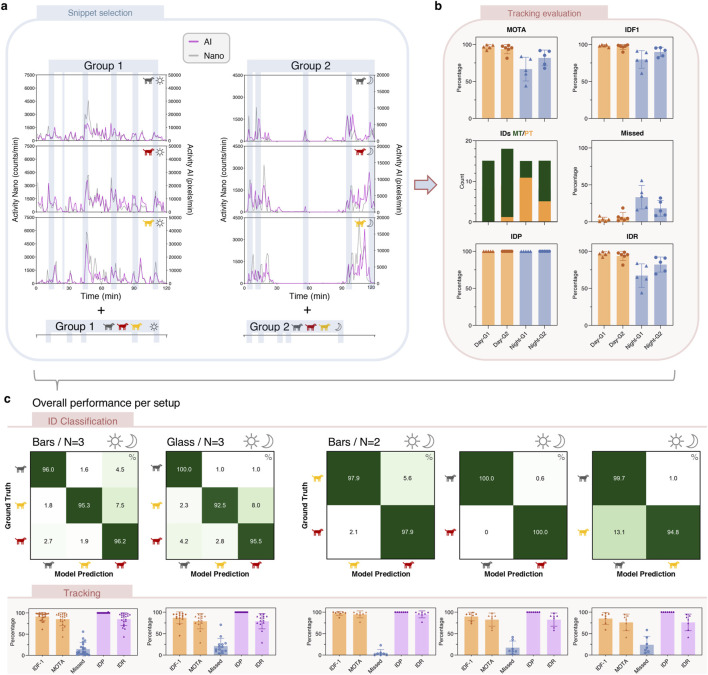
Field validation of reID and tracking. **(a)** To validate our tracking and reID models, several 5-min snippets (blue shading) were selected with variating activity levels and animal interactions across diverse conditions (different room setups, group sizes and day/night conditions). As illustrated here for the bars setup, snippets (blue bars) are selected from the 2-h actigram videos during day and night from all six animals, which were divided into two groups (G1 and G2). **(b)** Tracking metrics were calculated for each individual snippet and pooled per group and per condition (day/night) as mean ± SD with each symbol representing an individual video snippet. Tracking metrics for the bars setup (N = 3) were evaluated on a total of 39,748 frames giving 119,324 targets. IDs MT/PT: number of IDs that are Mostly Tracked (>80%, green)/Partially Tracked (20%–80%, orange). **(c)** Detailed tracking metrics per group and condition are merged to obtain overall tracking performance for our two room setups (bars and glass) and smaller group sizes of 2 animals. Overall tracking metrics are represented in mean ± SD with each symbol representing an individual video snippet. For glass (N = 3), 26,225 frames were analyzed giving 78,679 targets. For bars (N = 2), ∼11,300 frames were analyzed per harness combination giving a total of 67,878 targets. Horizontal rows in the confusion matrices may sum to over 100% due to the occasional assignment of double IDs (one correct and an additional wrong ID).

After normalization of both different metrics, 2-h actigrams for both activity measurements were visually very similar for all animals, across a range of activity levels (Figures 4b, 5a). During daytime, normalized accelerometer values were sometimes “larger” compared to AI. These generally corresponded to periods of excitement in which animals were jumping or rubbing on the floor. As the accelerometer incorporates movements over x, y and z-axes, these short, intense movements resulted in very high counts while the actual “distance moved” was much smaller. As our AI activity tracking uses a uniform pixels-based measurement irrespective of the intensity, it resulted in a more objective representation of the actual “distance moved”. For linear movements (animals walking or running), there was a perfect overlay of activity read-outs of both techniques upon normalization. During the night, AI activity patterns were still highly similar to the accelerometer but showed more differences due to imperfect bounding boxes (night is a more challenging scenario, see also Figure 3a) and occasional ID switches. Similar to daytime, AI activity tracking more accurately represented the ‘distance moved'.

The similarity in the two methods was emphasized by a strong correlation between AI and accelerometer activity outcomes across all time spans: r = 0.8825 for 1-min intervals which improved further to 0.9257 and 0.9650 for 15 min and 2 h respectively (Figure 4b).

Along with detailed actigrams, it is useful to have a more general overview of the animals’ activity by classifying the time spent as either mobile (moving from one point in space to another) or immobile (remaining in the same location). In single-housed animals, our AI model achieved an excellent classification of both mobile (96.8%) and immobile (94.0%) occurrences (Figure 4c, left panel). Most errors occurred in immobile classifications, due to imperfect bounding boxes or bounding boxes changing shape without animals actually moving, e.g., when tail wagging or heads moving while remaining in the same location. Equally, in group-housed animals (N = 3), we reached a very good classification accuracy despite this challenging setting: 90.4% for immobile and 81.0% for mobile (Figure 4c, left panel). The drop in performance was primarily due to imperfect bounding boxes when animals cross each other and/or at night.

#### 3.4.2 Field validation of reID and tracking

While the impressive results described above already imply strong reID and tracking, we manually assessed their performance in detail on several 5-min snippets as described in the Methods section and illustrated for bars setup in Figure 5a. For the latter during daytime, all tracking metrics were close to perfect, reflected in 32 of 33 total IDs being mostly tracked (>80% of their lifespan). At night, there were more missed detections due to the challenges of recognizing animals in greyscale colors and their tendency to cluster together while sleeping. This is reflected in lower MOTA and IDR scores, and 16 out of 30 IDs being partially tracked. However, IDP scores remained high, indicating that when an animal was detected, the correct ID was assigned most of the time (Figure 5b).

The small differences in reID performance between the two evaluated groups (N = 3 each) are attributed to chance and individual behavior differences, e.g., whether they like being close to each other and their preference where to stand, sit or rest. Merging results for both groups in the bars setup across day and night yielded an excellent overall reID accuracy of 95.3%–96.2% and average scores of 85.1% MOTA, 91.4% IDF1, 100% IDP, 85.3% IDR and 14.9% missed detections (Figure 5c).

For the setup with glass fronts and group sizes of N = 3, we achieved a similar outstanding reID accuracy ranging from 92.5% to 100% across day and night footage. Also tracking metrics were similar, with high IDF1 (87.0%) and IDP (100%) scores; and occasional missed detections (21.0%) which are reflected in the MOTA (79.0%) and IDR (79.9%) scores (Figure 5c).

In the assessment of reID and tracking performance for three harness combinations with group sizes of N = 2, all combinations achieved at least 94.8% reID accuracy across day and night. The R-N/Y-St combination proved to be superior with the fewest missed detections (5.1%) and highest scores across all metrics (94.9% MOTA, 97.2% IDF1, 100% IDP and 94.9% IDR) (Figure 5c).

#### 3.4.3 Field validation of pose classification

Our pose classifier achieved an excellent recognition of Standing (97.4%) and Standing Up (94.6%) when implemented in the pipeline, followed by Standing Down (86.1%). Lying and Sitting classes were the least performant with accuracies of 70.4% and 66.5% respectively. Lying was quite often confused with Standing, and Sitting was mixed up with Lying and Standing (Figure 4c, right panel).

#### 3.4.4 Field validation of behavioral classification

**FIGURE 6:**
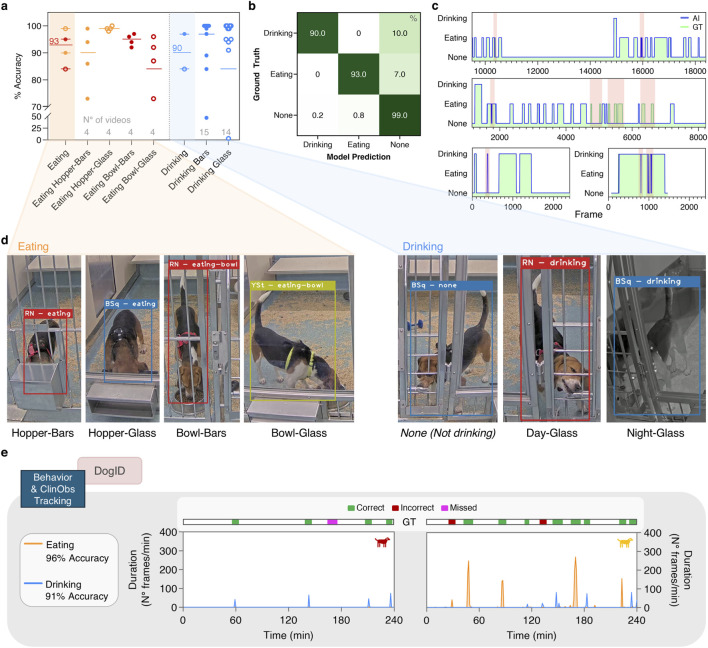
Field validation of Behavioral Classifier. **(a)** Overview of the frame-level accuracies for eating and drinking classes. The orange and blue shaded areas correspond to the overall mean accuracies which were calculated by summation of the different eating and drinking conditions listed in the y-axis. **(b)** Confusion matrix with frame-level accuracies (in %) of our behavioral classifier when implemented in the pipeline. **(c)** Frame-by-frame overlay of model predictions (AI) and ground truth (GT) annotations for five representative fragments. Red shaded areas highlight mismatches. **(d)** Representative screenshots of the different eating and drinking conditions, taken from analyzed video fragments **(e)** Real-life implementation of our AI model pipeline on 4 h food-access footage for two representative animals, with ground truth assessment in the top bar. To enable visualization, framewise AI predictions were binned per minute for each behavioral class. Class accuracies were calculated on event-level over ten animals.

Our behavioral classifier demonstrated excellent frame-level performance when implemented in the pipeline (Figure 6b), similar to the test set (Figure 3e). For *eating*, we assessed four different conditions (Figure 6d) where *Eating hopper-glass* consistently excelled across all fragments, followed by *Eating Bowl-Bars* (Figure 6a). The *Hopper-Bars* and *Bowl-Glass* conditions showed more variation between different fragments. However, the lowest-performing video still achieved 73% frame-level accuracy in both conditions. Overall, *eating* reached an outstanding frame-level accuracy of 93% (Figures 6a,b).

*Drinking* was in general excellently detected for both glass and bars during day and night, with at least 84% accuracy and often reaching 100% (frame-level). Only two snippets showed low accuracy of 47% and 3% (Figure 6a) due to the animal pausing frequently when drinking and an inadequately predicted bounding box that did not include the animal’s head, respectively. Across all snippets, *drinking* showed an excellent frame-level accuracy of 90% (Figures 6a–d).

Finally, the background class *none* achieved a frame-level accuracy of 99% (Figure 6b), with incorporation of the challenging snippets comparable to the two behaviors (*not eating* and *not drinking*), again indicating that our model generates a low number of false positives. Similar to our test set, the model never mixed-up *eating* and *drinking*; misclassifications were limited to confusion between the behavior and the background class (*none*). Figure 6c also illustrates the near-perfect overlay of our AI predictions on the manual ground truth at frame level.

In real-life situations, accurate frame-by-frame predictions are less critical as long as overall events are detected. Analysis of continuous 4 h food-access footage from ten animals with different eating patterns (2 representative examples shown in Figure 6e) revealed high event-level accuracy of 96% for *eating* and 91% for *drinking*, with minimal missed or incorrect detections.

#### 3.4.5 Field validation of ClinObs classification

**FIGURE 7:**
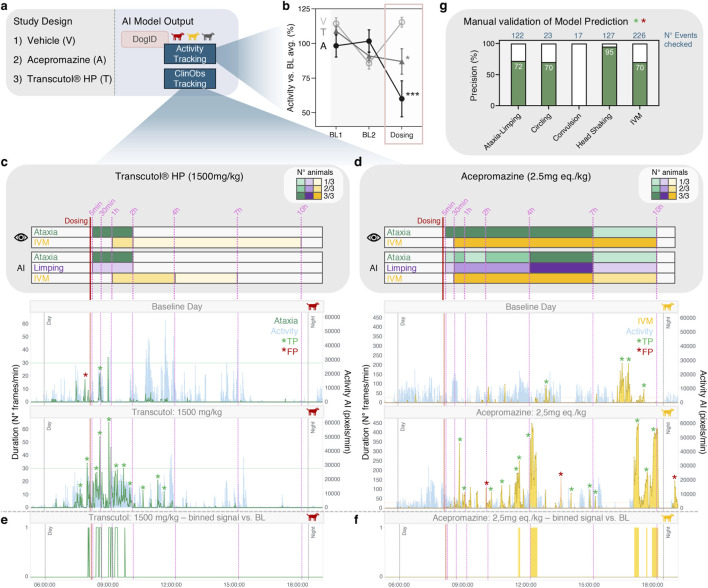
Field validation of ClinObs Classifier. **(a)** Study Design: 3 groups consisting of 3 animals/group, dosed with either Vehicle P.O. (H_2_O, V), Acepromazine 2.5 mg. eq/kg P.O. **(a)** or Transcutol® HP 1500 mg/kg P.O. (T). **(b)** Evolution of 24 h activity (mean ± SD) across the baseline and dosing days for the different treatment groups. Activity is represented as percentage normalized to the average baseline activity of the corresponding animal. Statistical analysis included an ANOVA on the percent difference relative to the baseline mean, followed by t-tests comparing each treatment with the vehicle, using a Dunnett correction for multiple comparisons. **(c)** Schematic representation of the *in person* observations and AI model detections across the different time periods post-dosing for the Transcutol® HP group. A representative, framewise AI model output is shown for ataxia in one animal on a baseline day and the dosing day. Significant and/or aberrant AI signals that were manually checked are marked with a green (true positive, TP) or red (false positive, FP) asterisk. **(d)** Schematic representation of the *in person* observations and AI model detections across the different time periods post-dosing for the Acepromazine group. A representative, framewise AI model output is shown for IVM (involuntary muscle movements) in one animal on a baseline day and the dosing day. Significant and/or aberrant AI signals that were manually checked are marked with a green (true positive, TP) or red (false positive, FP) asterisk. **(e)** Remaining ataxia signals (per minute) on the dosing day after applying an adaptive baseline thresholding strategy to eliminate the baseline signals. **(f)** Remaining IVM signals (per minute) on the dosing day after applying an adaptive baseline thresholding strategy to eliminate the baseline signals. **(g)** Manually assessed event-level precision of significant/aberrant AI detections.

We performed a validation study with two reference compounds (Acepromazine 2.5 mg eq./kg P.O. and Transcutol® HP 1500 mg/kg P.O.) and one control group (water) to validate our AI pipeline’s ability to detect clinical signs in a realistic experimental setting (Figure 7a). For this, we investigated whether our model could identify the same behavioral changes and ClinObs as noted during the standard *in person* observations.

Transcutol® HP is a commercially available vehicle that induces ataxia and IVM (in canines), when used in its pure form (internal data). Our model accurately detected ataxia in all animals within the same period as the *in person* observations. In one animal, limping was also occasionally assigned together with ataxia (Figure 7c). When implementing our adaptive baseline thresholding strategy to filter out baseline noise and highlight significant changes on the dosing day, clear *Ataxia* signals remain within the expected time window (Figure 7e). In addition, our model detected *IVM* in the same two animals as the *in person* observations with a slight difference in IVM duration (Figure 7c).

Acepromazine is a known sedative that induces unstable gait (ataxia-like) and IVM (in canines)(VCA-Animal-Hospitals, 2023). Our AI activity tracking successfully detected the decreased activity observed during *in person* assessments in these animals compared to baseline (Figure 7b). Despite the sedative effect with only short periods of movement, our model successfully identified the ataxia-like gait as either *Ataxia* or *Limping* in all animals, during the majority of the expected time frame (Figure 7d). As they are both phenotypes of abnormal gait, AI outputs for *Ataxia* and *Limping* signals were merged together for the manual analysis to boost the model’s performance and enable better differentiation compared to the baseline noise. When plotted together with the animals’ activity, true positive signals (high abnormal gait-to-low activity) could be clearly distinguished from the baseline noise (low abnormal gait-to-high activity) (Supplementary Figure S3). Furthermore, our model successfully detected the IVM in all animals within the expected time window with exception of one animal where the AI detections were shorter in duration compared to *in person* observations (Figure 7d). Upon implementing our adaptive baseline thresholding strategy, clear IVM signals remain, although fewer in number as (also correct) baseline events are filtered out (Figure 7e).

A control group was included to investigate whether this mock-treatment would result in observable differences in AI signals after dosing compared to baseline. While our model correctly detected IVM and ataxia-like signals in some animals on the treatment day, the AI signal patterns were highly similar compared to baseline (Supplementary Figure S2A,B). Similarly, applying our thresholding strategy resulted in almost no notable signals (Supplementary Figure S2C,D), again showing that our model can identify actual treatment-related observations.

As final part of the field validation, we performed a manual video analysis for a large number of substantial AI signals and/or patterns to evaluate the precision of our model detections on event-level (Figure 7g), opposed to the framewise precision on the test set (Figure 3f). As mentioned above for the Acepromazine-dosed group, we pooled ataxia and limping detections for this analysis as an *abnormal gait* phenotype. For almost all model detections in the study, we showed strong to excellent precisions: circling (70%), IVM (70%), ataxia-limping (72%) and head shaking (95%). While convulsion detections showed 0% precisions, it is important to note that in many detections, the animal was rubbing (35%) or showing ataxia (6%); which can be highly comparable to an actual convulsion.

## 4 Discussion

To enable continuous monitoring of activity, behavior and abnormalities (ClinObs) during non-clinical safety studies in canines, we developed and implemented a novel video-based AI model pipeline composed of multiple interconnected ANNs. This pipeline integrates a detector, an identification module and a tracker module linking detections from the same animal across different frames together in a single track that is fed into dedicated pose, behavior and ClinObs classifiers (Figure 1). The identification module plays a critical role in enabling single- and group-housed conditions, according to animal welfare standards during continuous monitoring.

With our unique approach, models receive only image/video data as input, no other sensors are required, as they are completely pixel appearance-based, in contrast to the commonly used skeleton/keypoint based models. This approach enables recognition of various behaviors and visually subtle ClinObs that would not be discernible when relying on keypoint data alone. Furthermore, the approach offers high flexibility, supporting training for diverse observations and could accommodate a variety of camera configurations, including non-top-down viewpoints involving occlusions.

Given that appearance-based models require extensive data to achieve robustness–particularly for detecting subtle behavioral features–we optimized our labeling process by employing a single-dot annotation strategy that allowed efficient labeling of over 1.8 million frames.

A key aspect in applying computer vision techniques in practice is translating performance metrics, obtained from initial test sets using short minute-length video snippets, into real-word scenarios involving hours or even days of footage. The initial test set often represents a limited part of the data set (in our case ∼20–25%) that can never fully represent the variation present in real-life situations. Therefore, our work included an extensive field validation of our ANNs on hours of footage, corroborating the results observed on our test sets.

Our first key achievement is the successful implementation of multi-animal identification and tracking over multiple often-occluded camera feeds, made robust by utilizing colored harnesses with distinct reflective patterns. This approach enabled long-term individual animal tracking within groups, enabling downstream model outputs (poses, activity, behaviors and ClinObs) to be attributed to specific animals without compromising welfare-related social interactions or spacing requirements. We have implemented the harnesses in several studies up to 4 weeks without any irritation or impact on the skin or fur of the animals (internal data). To achieve performant tracking, we implemented the Jonker-Volgenant algorithm on weighted scores of the detector, ID classifier and IoU. When comparing the same group size (N = 3) and setup (bars), tracking metrics were nearly identical between test set and field validation (Figures 3c, 5). This outstanding performance was reflected in the excellent correlation between our AI model and the accelerometer (Figure 4) - even in the most challenging situations of group-housed animals and at night. Moreover, our AI activity tracking resulted in a more objective representation of the actual ‘distance moved’. As missed detections for longer periods of time (exceeding some frames) only occurred when animals were immobile and at night (accelerometer values close to or zero, typically when sleeping), they did not interfere with accurate activity tracking. Their downstream impact is also less of a concern since most behaviors and ClinObs occur in mobile animals or include at least some movement. However, clinical signs that occur in truly sedentary animals (e.g., IVM) are at risk to be missed when they occur at night.

Our pose classifier performed similarly for Standing and Standing Up across the test and field validation set. For Lying and Sitting the performance dropped during the field validation while for Standing Down the performance increased (Figures 3d, 4c). These differences in accuracy are likely due to insufficiently varied data in the test set–again highlighting the value of field validation. Like the tracking module, the behavioral classifier performed equally well on the test set vs. field validation: drinking 93.1% vs. 90.0%, eating 89.6% vs. 93.9% and none 95.4% vs. 99%, respectively. Event-level accuracies remained similar to those on frame-level, due to a small number of misclassified behaviors.

The disparity between top-1 (79.0%) and class (47.9%) accuracy of our ClinObs model underscores the class imbalance inherent in the dataset, reflecting that performance varied considerably across different observation types; linked to their representation in the training data. Observations like IVM also proved inherently difficult to classify as they are characterized by subtle visual cues. Furthermore, the prevalence of the background class *none* resulted in a higher precision relative to recall. While model predictions tend to be correct, the model exhibits caution in recognizing all ClinObs due to its preference for predicting *none*, which consequently results in a higher risk for false negatives. The occasional confusion between similar ClinObs classes is a potential source of error that, while not ideal, can be mitigated by the presence of a human reviewer. Crucially, the model’s robust detection of key signs such as circling (86.6%) underscores its potential to assist in non-clinical assessments.

As a final part, we validated our AI pipeline for the detection of clinical signs by using reference compounds for ataxia and IVM. While on the test set, the performance for these classes was suboptimal ranging from 30.1% (IVM) to 35.2% (limping) and 40.4% (ataxia); our AI model succeeded in detecting the *in person* observed ataxia and IVM in all animals within the expected time frame. In contrast to the clear Transcutol® HP-induced ataxia, Acepromazine-treated animals showed more of an ataxia-like, unstable gait phenotype. This explained why our model more often confused ataxia with limping in these animals, while this rarely happened in the Transcutol® HP group. For the analysis, *Ataxia* and *Limping* signals were therefore merged. While our model’s inability to distinguish between these two classes in certain phenotypes is a definite limitation, there will always be a need for a human reviewer (at least for the foreseeable future) who can mitigate and assign the correct classification.

Overall, we demonstrated a consistent, high performance of our AI pipeline in real-word scenarios, both on our fully annotated test sets, as well as in extensive field studies. Our activity and behavioral tracking modules are highly reliable, and even for subtle ClinObs, we showed very promising results and were able to detect clinically relevant observations amongst the model-generated background.

This can significantly improve not only drug safety evaluation, but also the refinement of these studies by continuous animal welfare and health monitoring. It aligns perfectly with the FDA Modernization Act 2.0 that focuses on the use of new approach methodologies (NAMs) and implementation of AI (Zushin et al., 2023) where the aimed reduction of animal numbers emphasizes the importance to extract the most possible information out of every performed *in vivo* study. We believe that AI-video monitoring should become a standard practice in the coming years to map out detailed *in vitro-in vivo* correlations. To our knowledge, we are the first to develop such a robust and integrated AI pipeline for canines aimed at preclinical research purposes. As our proposed AI pipeline is designed to be generic, it should allow for application for diverse observations across camera setups and species after sufficient retraining. In addition, our identification strategy using a clear visual identifier can be easily transferred to a number of non-rodent species, such as minipigs or non-human primates.

In our future research, we aim to enhance pose classification and validate it in group-housed conditions; and further develop our behavior and ClinObs classifier model. This includes augmenting underperforming classes with more data and introducing new classes. Since this process will take time, we plan to boost the performance of our model in the meantime by visually combining outputs of several modules. For example, a *Standing* pose that persists for an extended period, combined with low activity and high immobile time, could indicate neurological symptoms such as abnormal posture or catalepsy. Similarly, AI model output can be combined across similar classes, as illustrated for *Ataxia* and *Limping* in Acepromazine-treated animals (Supplementary Figure S3). When adding additional information on the animals’ activity, signals can be identified that warrant further manual investigation, such as high *Abnormal gait* detections compared to low animal movement. Additionally, we plan to explore anomaly detection techniques to improve detection of rare and novel, unseen ClinObs, as well as few-shot techniques to cover the entire long-tailed training data distribution range.

In conclusion, our proposed pixel appearance-based computer vision AI approach holds tremendous potential to significantly impact the field of preclinical and behavioral research. It can provide a complete and detailed mapping of individual animal behavior which enables the investigation of behavioral patterns and premonitory signs, and a true quantification of spontaneous occurrences compared to on-study events. This significant improvement in observational accuracy will enhance our understanding of drug safety and as a NAM, contribute to the refinement of preclinical *in vivo* studies through the application of the 3Rs principle (reducing, refining, and replacing the use of animals). Moreover, it underscores a commitment to animal welfare and health monitoring, ensuring a positive contribution to ethical research practices.

## Data Availability

The datasets presented in this article are not readily available because they are proprietary to Johnson and Johnson. These can be obtained from the corresponding author on reasonable request and upon permission of Johnson and Johnson. Requests to access the datasets should be directed to ikopljar@its.jnj.com.

## Supplementary material

SUPPLEMENTARY FIGURE S1 Comparative analysis supporting Detector ANN and harness selection. **(A)** Test set performance (F1) of all object detectors we compared and their IoU with the manually annotated bounding boxes. YOLOv2 was selected to maximize F1 and maintain satisfactory IoU. **(B)** Confusion matrix of our 6-ID model, based on which Black-Dots and Grey-Fluo were discarded.

SUPPLEMENTARY FIGURE S2 AI Model output for vehicle animals in Field validation study. **(A)** A representative, framewise AI model output for ataxia in one vehicle-treated animal on a baseline day and the dosing day. Significant and/or aberrant AI signals that were manually checked are marked with a green (true positive, TP) or red (false positive, FP) asterix. **(B)** A representative, framewise AI model output for IVM (involuntary muscle movement) in the same vehicle animal on the same days. Significant and/or aberrant AI signals that were manually checked are marked with a green (true positive, TP) or red (false positive, FP) asterix. **(C)** Remaining ataxia signals (per minute) on the dosing day after applying an adaptive baseline thresholding strategy to eliminate the baseline signals. **(D)** Remaining IVM signals (per minute) on the dosing day after applying an adaptive baseline thresholding strategy to eliminate the baseline signals.

SUPPLEMENTARY FIGURE S3 Merging ClinObs classes to improve model performance. **(A)** A representative, framewise AI model output for ataxia in one Acepromazine-treated animal on a baseline day and the dosing day. **(B)** A representative, framewise AI model output for limping in the same Acepromazine-treated animal on the same days. **(C)** Framewise AI model output when merging ataxia and limping detections. Significant and/or aberrant AI signals that were manually checked are marked with a green (true positive, TP) or red (false positive, FP) asterix.
