# Supplementary figures and images for: Appearance-based computer vision pipeline for multi-animal monitoring of canine activity, behavior and clinical observations
Source: Front Toxicol. 2026 Feb 18;8:1758963. doi: 10.3389/ftox.2026.1758963 (PMC12957207; doi:10.3389/ftox.2026.1758963)

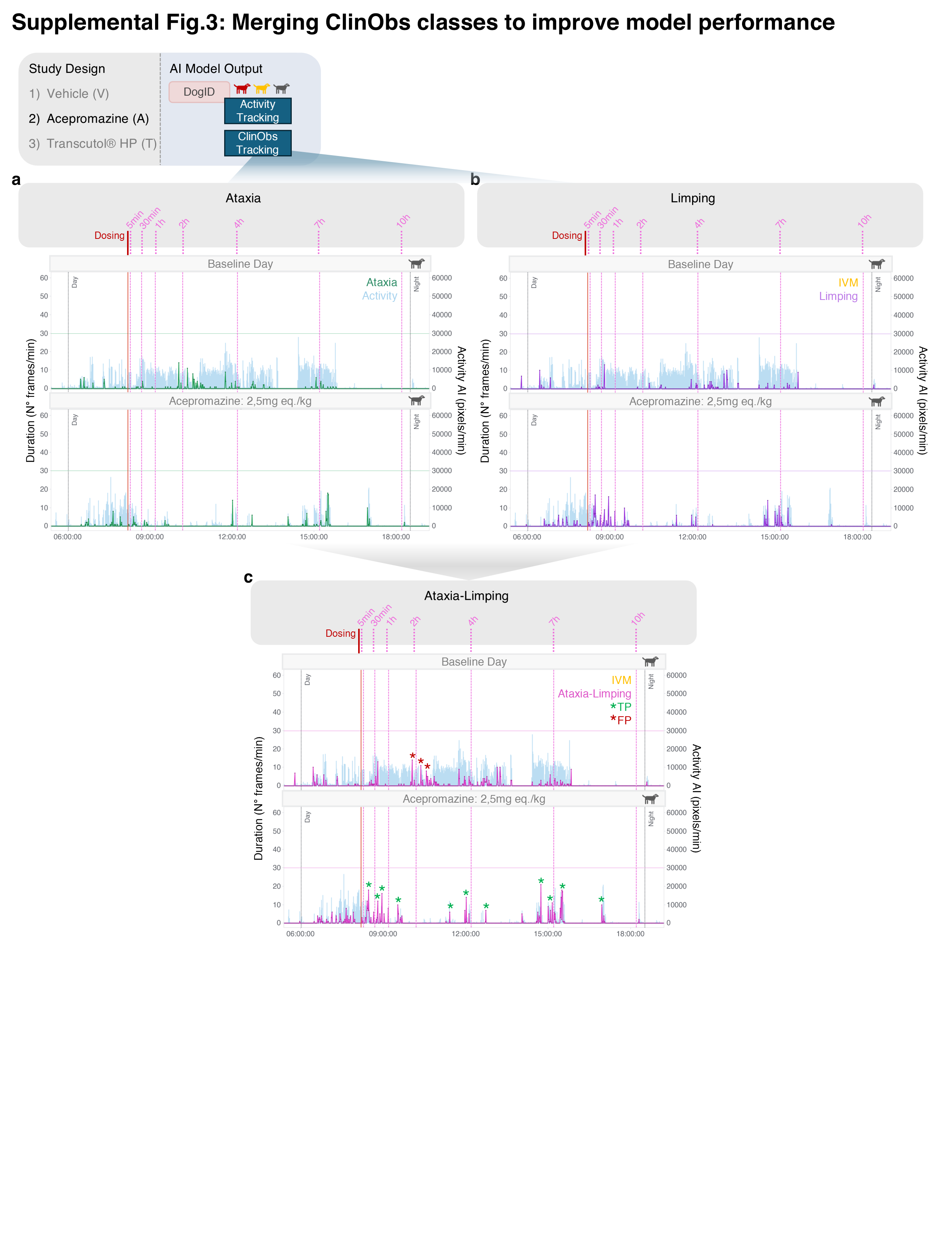

Supplement: Supplementary file 1 [file Image3.tif]

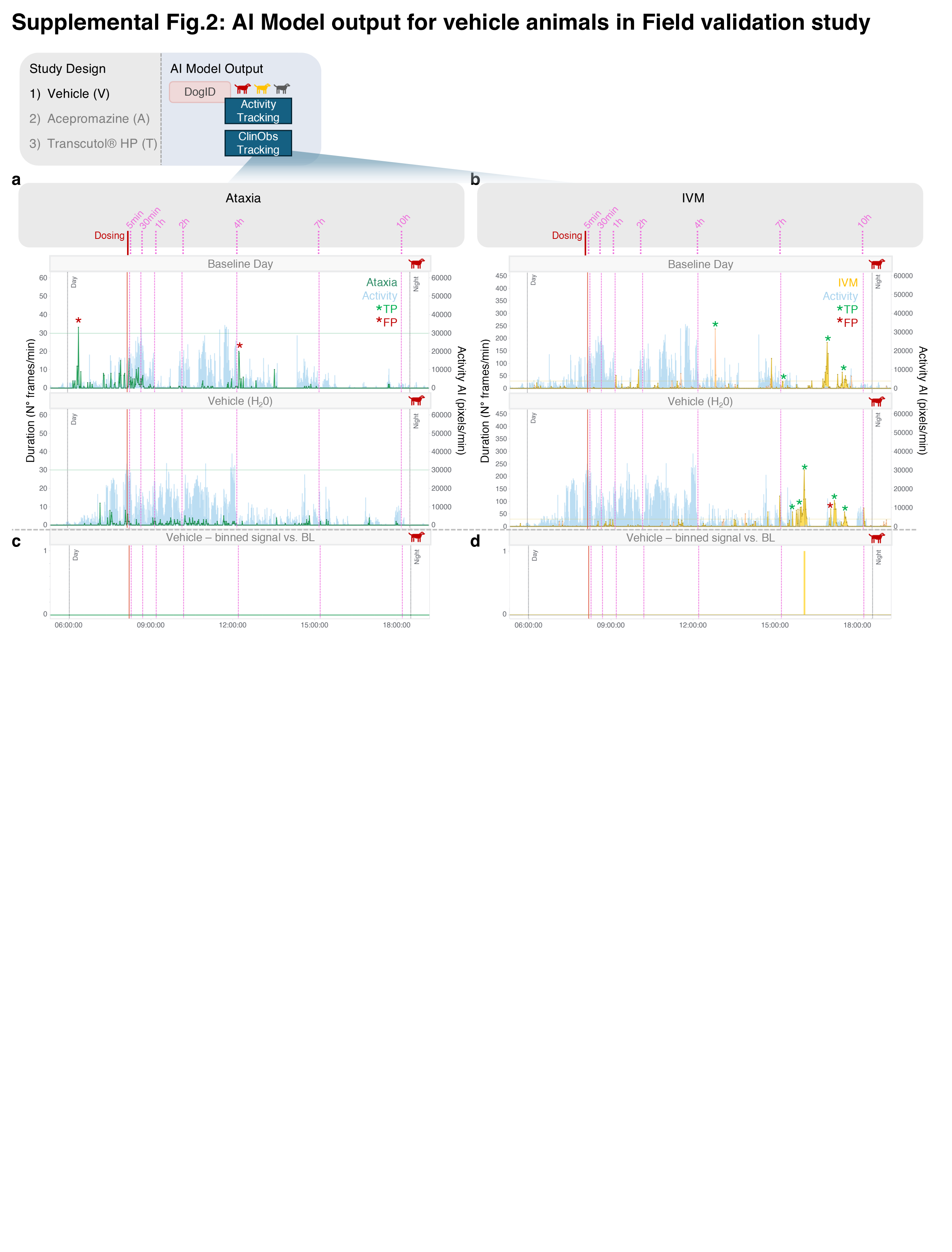

Supplement: Supplementary file 2 [file Image2.tif]

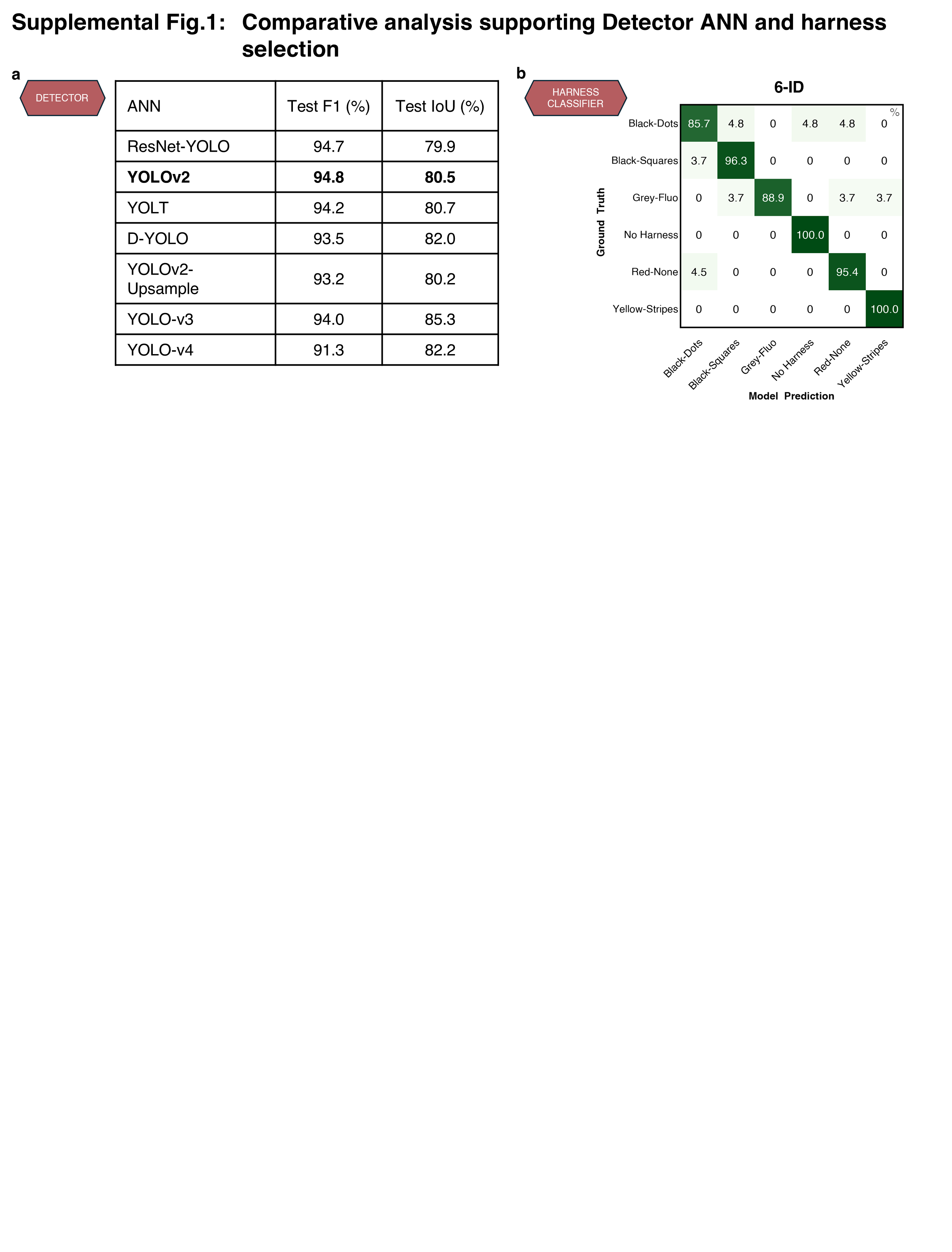

Supplement: Supplementary file 3 [file Image1.tif]
